# The impact of disease characteristics on multiple sclerosis patients’ quality of life

**DOI:** 10.4178/epih.e2017008

**Published:** 2017-02-19

**Authors:** Aziz Rezapour, Abdollah Almasian Kia, Sahar Goodarzi, Mojtaba Hasoumi, Soraya Nouraei Motlagh, Sajad Vahedi

**Affiliations:** 1 Health Management and Economics Research Center, Iran University of Medical Sciences, Tehran, Iran; 2 Department of Health Economics, School of Health Management and Information Sciences, Iran University of Medical Sciences, Tehran, Iran; 3 Department of Health Economics, School of Management and Information Sciences, Shiraz University of Medical Sciences, Shiraz, Iran; 4 Student Research Committee, Shiraz University of Medical Sciences, Shiraz, Iran; 5 Public Health Department, School of Health and Nutrition, Social Determinants of Health Research Center, Lorestan University of Medical Sciences, Khorramabad, Iran; 6 Student Research Committee, Zabol University of Medical Sciences, Zabol, Iran

**Keywords:** Quality of life, Multiple sclerosis, Iran, Disease attributes

## Abstract

**OBJECTIVES:**

The aim of this study was to assess the quality of life (QoL) of patients with multiple sclerosis (MS), and to investigate the effects of characteristics of MS such as disease course, severity, and relapses on patients’ QoL.

**METHODS:**

This was a cross-sectional study, in which 171 patients were enrolled. Health-related QoL was assessed using the Persian version of the Multiple Sclerosis Quality of Life-54 questionnaire. To measure patients’ disability status, we used the Expanded Disability Status Scale. Other variables included in the study were disease course and relapses of the disease.

**RESULTS:**

The average scores for patients’ physical and mental QoL were 60.9±22.3 and 59.5±21.4, respectively. In a bivariate analysis, disease course, severity of the disease, and relapses were significantly associated with the physical and mental health composite scores. In a hierarchal regression analysis, disease course, severity of the disease, and relapses were responsible for 38 and 16% of the variance in physical and mental QoL, respectively. It was also observed that relapses were a strong predictor of both physical and mental QoL.

**CONCLUSIONS:**

Our results showed that disease characteristics significantly affected both dimensions of QoL. It is therefore suggested that health care providers should be aware of these characteristics of MS to more successfully improve MS patients’ QoL.

## INTRODUCTION

Multiple sclerosis (MS) is one of the most widespread neurological diseases in young adults, and affects approximately 2.3 million people worldwide. The disease most commonly appears in people aged 20 to 40 years, the age group considered to be the economically active population [1]. MS, after stroke and before Parkinson disease, is considered to be the second most debilitating chronic disease of the central nervous system [2,3]. MS symptoms include impaired coordination and balance, muscle cramps, fatigue, pain, and visual disturbances, and these symptoms can lead to restricted mobility and, in some cases, hospitalization [4,5]. Studies have found that patients suffering from MS experience a quality of life (QoL) lower not only than the general population, but also than those suffering from other chronic diseases [6,7]. Extensive physical disabilities, a lack of effective treatment methods, and the unknown causes of MS provide convincing evidence of the negative impact of this disease on patients’ QoL [8]. As MS progresses, patients encounter new manifestations of the disease, with more limitations in their daily activities and working abilities. As the disability of patients increases, they become dependent on their family for carrying out their daily routines and activities, which leads to a reduction of their QoL [9].

The concept of QoL in the health field, which is based on patients’ individual perspectives, appeared following the development of technologies that increased longevity, and is used as a consideration in the assessment of social policy-making and health care outcomes [10]. Measuring QoL can be a good definition of treatment success, the improvement of a patient’s health status after an invasive intervention, and the overall effect of treatment from a patient’s perspective [11].

In the field of medicine, researchers often use the concept of health-related quality of life (HRQoL), which specifically assesses the impact of a disease or its treatment on an individual’s self-conception of health status and life satisfaction. MS patients’ QoL is often measured by the Multiple Sclerosis Quality of Life (MSQoL)-54 questionnaire, which is considered to be the most common and standardized disease-specific instrument for the assessment of QoL of MS patients [12,13].

Improvements can be made in the QoL of MS patients only through understanding effective, behavioral, mental, and social factors [14]. Therefore, studying the QoL of MS patients would help health authorities and policymakers in planning and implementing interventions to increase MS patients’ QoL [15]. This study aimed to assess the effect of disease characteristics such as severity, disease course, and relapses in the past three months on the QoL of MS patients.

## MATERIALS AND METHODS

This was a cross-sectional study carried out among MS patients referred to the Center for Special Diseases and Multiple Sclerosis Society of Shiraz between February and October 2013. The inclusion criteria were age greater than 18 years and having at least a 1-year history of MS. Patients who were not willing to participate in the study or those who had a concomitant chronic disease that would impact their QoL were excluded. Ultimately, 171 patients enrolled in the study and were asked to fill out a questionnaire containing demographic (age, sex, marital status, educational level, and employment status) and clinical (disease duration, relapses during in last three months, and age of onset) questions. Disease course and neurologic disabilities for each patient were measured by a neurologist and recorded by an interviewer. All patients were classified as relapsing-remitting, primary progressive, or secondary progressive based on their disease course. To measure patients’ disability status, we used the Expanded Disability Status Scale (EDSS). The Kurtzke EDSS [16] is a quantitative method for assessing MS disability that provides a total score on a scale ranging from 0, indicating a normal neurological examination, to 10, indicating death due to MS. Based on their score, patients were categorized in three groups: mild disability (EDSS, 0.0 to 3.5), moderate disability (EDSS, 4.0 to 6.5), and severe disability (EDSS, 7.0 to 9.5).

HRQoL was assessed using the MSQoL-54 questionnaire. This questionnaire is a psychological instrument consisting of 54 standard questions measuring the QoL in MS patients. The MSQoL-54 questionnaire contains 36 questions in a short form for assessing overall health status and general QoL, and 18 questions designed especially for MS patients, with scales for health-related discomfort (4 items); sexual function and satisfaction (5 items); overall QoL (2 items); cognitive function (4 items); and energy, pain, and their social situation (3 items). The MSQoL-54 questionnaire assesses health status in two general scales: physical health (39 items) and mental health (15 items). The subscales of physical health include: physical function (assessing possible activities during a normal day), health perceptions, physical role limitations, pain, sexual function, social function, energy, and health distress. The five mental health subscales are as follows: overall QoL, emotional well-being, emotional role limitations (assessing any mental health related problems in working or any other daily activities, such as depression or anxiety), cognitive function (corresponding to problems related to focusing and thinking), and health distress (assessing weight loss and discouragement due to health problems). To calculate the desirability scores for each scale, the weighted average score of the constituent subscales are calculated. These scores range from 0 to 100; in each scale, a higher score means a better QoL.

In this study, we used the Persian version of the MSQoL-54 questionnaire, which was successfully validated by Ghaem & Haghighi [17], with a Cronbach alpha coefficient of 0.962 for measuring a patient’s QoL.

### Statistical analysis

Descriptive statistics such as mean, standard deviation (SD), number and percent were used to analyze all demographic and clinical variables, as well as the physical and mental dimensions of QoL. The Student t-test was used to compare mean differences in the physical and mental health composite scores by sex (male vs. female), marital status (single vs. married), education level (primary vs. secondary or higher), employment status (unemployed vs. employed), disease duration (≤ 5 years vs. > 5 years), age of onset (≤ 30 years vs. > 30 years), relapses in the past three months (yes vs. no), disease severity (mild vs. moderate-severe), disease course (relapsing-remitting vs. progressive). The Pearson correlation test was used to assess the relationship between the physical and mental health composite scores and continuous variables, such as disease duration, EDSS scores, and age of onset. To determine the predictors of the physical and mental health composite scores, hierarchical lineal regression was used. We entered two blocks of control variables and one block of predictors in the regression model. The control variables consisted of demographic variables including sex, marital status, education level, and employment status (model 1) and disease duration and age of onset (model 2). The predictors included disease severity, disease course, and relapses in the past three months. The beta coefficient (β), p-value, R-squared, adjusted R-squared, and F-value were reported for the hierarchical regression models. Data were processed and analyzed using Excel 2007 (Microsoft, Redmond, WA, USA) and Stata version 14.1 (StataCorp., College Station, TX, USA).

### Ethics

This study was approved by the Ethics Committee of the Shiraz University of Medical Sciences and written informed consent was obtained from all patients enrolled in the study.

## RESULTS

Table 1 presents descriptive statistics for the demographic and clinical characteristics of patients. The number (%) for dichotomous variables and mean (SD) for continuous variables were reported. A total of 171 patients (76.6% female) participated in this study, with a mean age of 35.7 (8.1) years. Statistically, there was no significant difference between males and females in terms of age (t_169_= 0.24, p= 0.8, t-test). Only 34.0% of patients were working; the rest were either unemployed or had to quit their job due to MS. The pluralities of participants had a primary education and were married. Approximately 83.6% of patients suffered from relapsing-remitting disease, while the remaining patients were categorized as having primary or secondary progressive disease.

**Figure 1.**
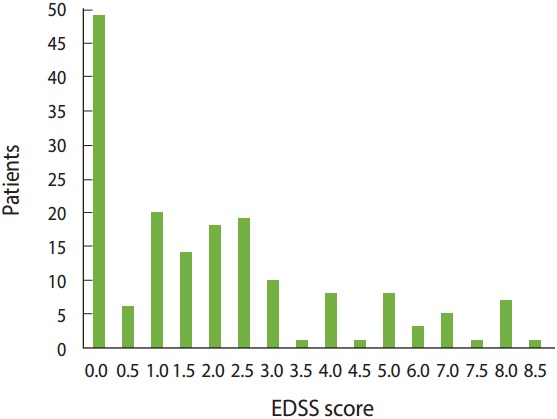
Distribution of patients by Expanded Disability Status Scale (EDSS) score.

The mean EDSS score was 2.1± 2.2, and 80.1% of patients had a mild disability (EDSS, 0.0 to 3.5), while 11.7 and 8.1% were categorized as having a moderate disability (EDSS, 4.0 to 6.5) and a severe disability (EDSS, 7.0 to 9.5), respectively. The distribution of patients by EDSS score is presented in Figure 1.

**Figure 2.**
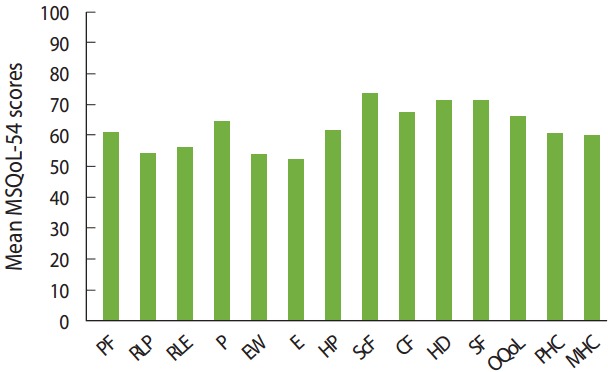
Mean scores of the Multiple Sclerosis Quality of Life (MSQoL)-54 subscales. PF, physical function; RLP, role limitation-physical; RLE, role limitation-emotional; P, pain; EW, emotional well-being; E, energy; HP, health perception; ScF, social function; CF, cognitive function; HD, health distress; SF, sexual function; OQoL, overall quality of life; PHC, physical health composite; MHC, mental health composite.

The average scores for patients’ physical and mental QoL were 60.9 ± 22.3 and 59.5 ± 21.4, respectively. According to the QoL subscale scores, social function and energy had the highest scores (74.1± 24.6) and the lowest scores (51.8± 21.2), respectively. Figure 2 presents the mean scores of the physical and mental subscales in our study.

Table 2 illustrates the relationships of the physical and mental dimensions of QoL with the demographic and clinical characteristics of patients. Females reported higher scores in the physical dimension than males (p< 0.05), while there was no significant difference in the mean mental composite score between males and females. Both physical and mental scores did not show statistically significant differences according to marital status, education level, or employment status.

Individuals suffering from relapsing-remitting disease had a significantly higher level of QoL in both the physical and mental dimensions. The relationship between QoL and disease relapse in the past three months was significant, such that patients who had experienced relapse had lower levels of physical and mental QoL than those who had not. Additionally, patients with an EDSS > 3.5, a disease duration of > 5 years, and an age of onset of > 30 years had the poorest QoL. These findings were also statistically significant.

Table 3 indicates that linear relationships were present between the dimensions of QoL and EDSS scores, disease duration, and age of onset. Based on the Pearson correlation test, EDSS scores showed a strong negative correlation with the physical health composite scores and a moderate negative correlation with the mental health composite scores. We observed a weak negative relationship between the physical dimension of QoL and disease duration, whereas the mental score did not show any association with the duration of disease. The correlation between age of onset and both composite QoL scores did not have statistical significance.

Hierarchical regression was used to assess the effects of demographic and clinical characteristics on the physical and mental health composite scores. As shown in Table 4, the total variance of the physical and mental dimensions that was explained by all variables was 0.382 and 0.166, respectively. After controlling for demographic variables (model 1), other variables, such as disease duration, age of onset, disease course, disease severity, and relapses in the past three months, were responsible for 35% of the variance in the physical composite score and 15% of the variance in the mental composite score. Disease severity and relapses in the past three months were the most significant determinants of physical status (p< 0.001), and relapses were the most significant predictor variable for the mental dimension.

## DISCUSSION

The aim of this study was to assess the effects of MS characteristics such as disease severity, disease course, and relapses in the past three months on the physical and mental dimensions of QoL in patients with MS. Our findings highlight that disease characteristics were especially important factors for the physical health composite score, more so than for the mental health composite score. Demographic and clinical characteristics explained 38% of the variance in the physical health dimension and 16% of the variance in the mental health composite score.

According to our results, there were no significant relationships between demographic factors and QoL in MS patients, except for sex and the physical health composite score. Some other studies conducted in Iran also reported that demographic characteristics were unrelated to HRQoL in MS patients [18-21]. In contrast, some studies showed that being female [22], having a lower education level [23], and being older [24] were factors related to poorer QoL.

In this study, we found a statistically significant negative relationship between the duration of disease and the physical dimension of QoL (r=-0.25, p< 0.001) which shows that a longer duration of the disease was associated with a poorer QoL. A negative relationship, but not a significant one, was observed between the duration of the disease and the mental dimension of QoL (r=-0.08, p= 0.25). Some studies have also stated that the duration of the disease negatively affected patients’ QoL [25-29]. However, other studies have reported contradictory results, with some indicating that there was no relationship between the duration of disease and QoL [24,30]. It seems that along duration of MS, with corresponding physical limitations, leads to a decrease in QoL in the physical dimension.

In the present study, patients with relapsing-remitting MS had better physical and mental QoL than patients with progressive MS. Mitchell et al. [15] showed that the disease course negatively affected patients’ QoL. The more aggressive the disease course was, the poorer the QoL. Tadić et al. [25] reported that since the disease develops faster in MS patients with a progressive disease course, their QoL is negatively affected.

The results of our study showed a negative relationship between the QoL score and severity of the disease, such that patients with less severe disease had a better QoL. In previous studies, a strong negative correlation between disability status and QoL has been reported [2,31-33]. Regression analysis showed that the severity of the disease was the most relevant independent variable predicting variance in the physical dimension of patients’ QoL. Szilasiova et al. [29] reported that disability was significantly related to both the physical and mental dimensions of QoL; but after adjusting the regression model to account for depression and anxiety, no significant relationship was observed between disability and the mental dimension of QoL. The contribution of disability level of MS to QoL variation has been reported to range from 2% in the US to 29% in Austria. This wide variation is due to the use of different questionnaires for assessing QoL [34]. Petersen et al. [35] argued that physicians mostly pay attention to the physical dimensions of a disease, while from the patients’ point of view; mental health is an important QoL determinant. Therefore, it should be noted that the EDSS is not able to provide information about the mental health dimension.

In the present study, it was also observed that relapse of the disease was a strong predictor of both physical and mental QoL. A study conducted by Miller et al. [36] showed that more relapses in the last two years were associated with poorer QoL. They also found that preventing disease relapse led to improvements in QoL. Jones et al. [37] also found that patients who experienced relapses had poorer HRQoL than those with no relapses. In another study, Mäurer et al. [38] showed that patients who had experienced severe relapses had significantly poorer HRQoL. Healy et al. [39] reported a relationship between relapses and HRQoL, in which patients with more severe relapses had a greater decline in HRQoL than those with mild relapses.

Our study has some limitations that should be considered. First, caution should be used in interpreting our findings as indicative of a causal relationship between QoL and disease characteristics because of the cross-sectional study design. Second, the sample size of participant was somewhat small. Third, we did not consider variables such as fatigue, depression, anxiety, and social support, which might also affect QoL.

Briefly, our study showed that both the physical and mental dimensions of MS patients’ QoL were significantly affected by disease characteristics, such as its severity and the occurrence of relapses. It is therefore suggested that health care providers should be appropriately informed about these characteristics of MS, in order to more successfully improve MS patients’ QoL.

## Figures and Tables

**Table 1.** Demographic and clinical characteristics of the patients

	Mean士SD	n (%)	Min	Max
Sex				
Male		40 (23.4)	0	1
Female		131(76.6)	0	1
Age (yr)	35.7±8.1		18	61
Marital status				
Single		70 (41.0)	0	1
Married		101 (59.0)	0	1
Education level				
Primary		118 (69.0)	0	1
Secondary or higher		53 (31.0)	0	1
Employment status				
Unemployed		113 (66.0)	0	1
Employed		58 (34.0)	0	1
Disease duration (yr)	7.6±4.6		1	24
Age of onset (yr)	28.1±7.6		13	50
Relapses in past 3 months				
Yes		106 (62.0)	0	1
No		65 (38.0)	0	1
EDSS score	2.1±2.2		0	8.5
Disease severity				
Mild		137 (80.1)	0	1
Moderate		20 (11.7)	0	1
Severe		14 (8.1)	0	1
Disease course			0	1
Relapsing-remitting		143 (83.6)	0	1
Secondary progressive		22 (12.8)	0	1
Primary progressive		6 (3.51)	0	1

EDSS, Expanded Disability Status Scale; SD, standard deviation; Min, minimum; Max, maximum.

**Table 2.** Mean physical and mental health composite scores by demographic and clinical variables

	Physical health	Mental health
Sex		
Male	54.37±3.89^*^	56.73±3.53
Female	62.90±1.86	60.40±1.84
Marital status		
Single	62.10±2.17	59.12±2.09
Married	59.18±2.76	60.15±2.63
Education level		
Primary	59.61±2.09	58.52±1.95
Secondary or higher	63.94±3.00	61.87±3.05
Employment status		
Unemployed	62.68±2.02	60.09±1.98
Employed	57.71±3.08	58.55±2.89
Disease duration (yr)		
≤5	66.01±2.72^**^	60.53±2.91
>5	57.70±2.14	58.91±1.94
Age of onset (yr)		
≤30	63.52±2.17^*^	61.94±2.11^*^
>30	56.21±2.69	55.20±2.50
Relapses in past 3 months		
Yes	53.85±2.07^***^	54.21±2.07^***^
No	72.41±2.36	68.22±2.30
Disease severity		
Mild	66.42±1.75^***^	62.18±1.79^***^
Moderate-severe	38.68±2.48	48.89±3.44
Disease course		
Relapsing-remitting	65.10±1.73^***^	61.75±1.74^***^
Progressive	39.48±3.40	48.24±4.01

Values are presented as mean±standard deviation.

* p<0.05,

** p<0.01,

*** p<0.001 by the Student t-test.

**Table 3.** Correlations between disease duration, age of onset, EDSS score, and health-related quality of life

Composite	Disease duration	Age of onset	EDSS score
Physical health	-0.259^***^	-0.128	-0693^***^
Mental health	-0.085	-0.140	-0.428^***^	

EDSS, Expanded Disability Status Scale.

*** p<0.001 by pair-wise Pearson correlations.

**Table 4.** Results of hierarchical regression assessing the effects of demographic and clinical variables on physical- and mental health composite scores

Variable	Physical health			Mental health		
	Coefficients			Coefficients		
	Model 1	Model 2	Model 3	Model 1	Model 2	Model 3
Sex						
Female	6.196	6.876	3.334	3.296	3.873	0.613
Marital status						
Single	2.744	9.526^*^	6.322	-0.970	3.142	1.277
Employment status						
Employed	-1.830	0.915	3.210	-0.370	1.514	2.607
Education level						
Secondary or higher	4.814	2.792	-0.002	2.857	1.597	-0.206
Disease duration (yr)		-1 436^***^	-0.416		-0.473	0.124
Age of onset (yr)		-0.668^**^	-0.470^*^		-0.486	-0.415
Disease course						
Progressive			-4.483			-5.066
Disease severity						
Moderate-severe			-15.085^**^			-3.101
Relapses in past 3 months						
None			14.734^***^			13.038^***^
Constant	53.750^***^	78.611^***^	73.690^***^	56.866^***^	70.982^***^	60.732^***^
n	170	170	170	170	170	170
F	1.55	4.58^***^	11.02^***^	0.42	1.08	3.55^***^
R-squared	0.036	0.144	0.382	0.010	0.038	0.166
Adjusted R-squared	0.012	0.112	0.348	-0.013	0.002	0.119

* p<0.005,

** p<0.001,

*** p<0.001 by hierarchical linear regression.
